# Morning Bright Light Treatment for Sleep-Wake Disturbances in Primary Biliary Cholangitis: A Pilot Study

**DOI:** 10.3389/fphys.2018.01530

**Published:** 2018-11-05

**Authors:** Matteo Turco, Nora Cazzagon, Irene Franceschet, Chiara Formentin, Giovanni Frighetto, Francesca Giordani, Nicola Cellini, Gabriella Mazzotta, Rodolfo Costa, Benita Middleton, Debra J. Skene, Annarosa Floreani, Sara Montagnese

**Affiliations:** ^1^Department of Medicine, University of Padova, Padova, Italy; ^2^Department of Surgery, Oncology and Gastroenterology, University of Padova, Padova, Italy; ^3^Department of General Psychology, University of Padova, Padova, Italy; ^4^Department of Biology, University of Padova, Padova, Italy; ^5^Chronobiology, Faculty of Health and Medical Sciences, University of Surrey, Guildford, United Kingdom

**Keywords:** liver, light, sleep, primary biliary cholangitis (PBC), circadian

## Abstract

Patients with Primary Biliary Cholangitis (PBC) exhibit delayed sleep-wake habits, disturbed night sleep and daytime sleepiness/fatigue. Such combination of symptoms is reminiscent of delayed sleep-wake phase disorder (DSPD), which benefits from morning light treatment. The aim of the present pilot study was to test the effect of morning light treatment in a group of 13 well-characterized patients with PBC [all females; (mean ± SD) 53 ± 10 years]. Six healthy individuals (4 females, 57 ± 14 years) and 7 patients with cirrhosis (1 female, 57 ± 12 years) served as controls and diseased controls, respectively. At baseline, all participants underwent an assessment of quality of life, diurnal preference, sleep quality/timing (subjective plus actigraphy), daytime sleepiness, and urinary 6-sulphatoxymelatonin (aMT6s) rhythmicity. Then they underwent a 15-day course of morning bright light treatment, immediately after getting up (light box, 10,000 lux, 45 min) whilst monitoring sleep-wake patterns and aMT6s rhythmicity. At baseline, both patients with PBC and patients with cirrhosis had significantly worse subjective sleep quality compared to controls. In patients with PBC, light treatment resulted in an improvement in subjective sleep quality and a reduction in daytime sleepiness. In addition, both their sleep onset and get-up time were significantly advanced. Finally, the robustness of aMT6s rhythmicity (i.e., strength of the cosinor fit) increased after light administration but *post-hoc* comparisons were not significant in any of the groups. In conclusion, a brief course of morning bright light treatment had positive effects on subjective sleep quality, daytime sleepiness, and sleep timing in patients with PBC. This unobtrusive, side-effect free, non-pharmacological treatment is worthy of further study.

## Introduction

Primary biliary cholangitis (PBC) is a rare, immune-mediated cholestatic liver disease (Selmi et al., 2011; Hirschfield and Gershwin, 2013; Beuers et al., 2015; Webb and Hirschfield, 2017), mostly affecting women, with the following features: cholestasis, serologic reactivity to antimitochondrial antibodies (AMA) or specific antinuclear antibody (ANA) reactivity, and histological evidence of chronic non-suppurative, granulomatous, lymphocytic small bile duct cholangitis. The disease is chronic and progressive, and can result in end-stage liver disease (Carbone et al., 2013; Lammers et al., 2014; Trivedi et al., 2016). From a clinical stand-point, the main features of the disease are the persistence of cholestatic abnormalities and symptoms including pruritus and fatigue. An association with extra-hepatic autoimmune conditions such as Sjögren syndrome and autoimmune thyroiditis is also frequent (Selmi et al., 2011; Hirschfield and Gershwin, 2013; Beuers et al., 2015).

Patients with PBC exhibit a delay in sleep-wake timing, which is associated with impaired sleep quality and quality of life (Montagnese et al., 2013). In addition, an interplay between their sleep-wake timing and the daytime course of symptoms such as pruritus and sleepiness has been documented (Montagnese et al., 2013). Thus a complex set of interacting and possibly conflicting factors simultaneously impinge on these patients' sleep and wake quality, and daytime symptoms such as sleepiness and fatigue (McDonald et al., 2010).

The combination of delayed sleep habits and impaired sleep quality is reminiscent of delayed sleep-wake phase disorder (DSPD), a circadian sleep-wake disorder characterized by considerable delays in sleep onset/wake-up times compared with the healthy population (Weitzman et al., 1981; Thorpy et al., 1988). The goal of DSPD treatment is to resynchronize the circadian clock timing system with the 24-h light-dark cycle: structured sleep-wake schedules and avoidance of exposure to bright light in the evening are advised. In addition, exposure to bright light shortly after waking up in the morning has been shown to advance sleep and melatonin timing (Weyerbrock et al., 1996; Revell et al., 2005).

The aim of the present study was to test the effect of a 15-day course of morning bright light treatment on sleep-wake and circadian variables in a group of well-characterized patients with non-cirrhotic PBC compared to healthy subjects and a group of patients with cirrhosis of etiology other than PBC.

## Patients and methods

Thirteen consecutive outpatients with PBC [all females; (mean ± SD) 53 ± 10 years] were enrolled. The diagnosis of PBC had been established based on the presence of at least two of the following criteria: AMA antibody or ANA specific antibody (gp210, sp100) positivity, abnormal alkaline phosphatase levels (at least 1.5 times the normal value), and/or a compatible liver histology (European Association for the Study of the Liver, 2017). None of the patients had advanced (histological stage IV) liver disease; median liver stiffness at the time of inclusion was 4.7 KPa (range: 3.8–8.1). All patients were on treatment with ursodeoxycholic acid on stable dose since PBC diagnosis (13–15 mg/kg/day; time since diagnosis: 6.7 ± 6.9 years).

Seven consecutive outpatients with cirrhosis (1 female, 57 ± 12 years) served as diseased controls. The etiology of cirrhosis was alcohol in 4 patients (including the only female) and viral in 3. Functionally, 4 were classified as Child's grade A, 2 as B, and 1 as C (Pugh et al., 1973).

Both patients with PBC and patients with cirrhosis were excluded if they could not comply with the study procedures, had misused alcohol in the preceding 6 months, had a history of head injury, cardiovascular/cerebrovascular disease or significant neurological/psychiatric co-morbidity (including pharmacologically uncontrolled dysthyroidism).

Six healthy volunteers (4 females; 57 ± 14 years) served as controls. None had a history, clinical or laboratory evidence of alcohol misuse, chronic liver disease or neurological/psychiatric disturbances, drank alcohol in excess of 20 g/day or took prescription medication.

None of the participants in any of the study groups was on chronic treatment with drugs known to affect sleep, daytime vigilance or melatonin production, had traveled across more than two time zones in the preceding 3 months or undertaken shift work in the preceding 5 years.

### Health-related quality of life

The 36-item short form health profile (SF-36) questionnaire was used to assess health-related quality of life. This generic, self-rated, health-related quality of life questionnaire provides an eight-scale profile, as well as two summary measures (SF-36 summary physical and SF-36 summary mental). The response to each question is scored, the eight profiles added and the data transformed into a scale of 0 (worst) to 100 (best) (Ware, 1992). The questionnaire does not include questions on sleep behavior.

### Sleep-wake assessment tools

The Horne-Östberg (HÖ) questionnaire was used to define diurnal preference as definitely morning (score 70–86), moderately morning (59–69), intermediate (42–58), moderately evening (31–41), and definitely evening (16–30) (Horne and Östberg, 1976).

The Pittsburgh Sleep Quality Index (PSQI) was used to assess night sleep quality over the preceding month. Questionnaire responses generate seven components each of which has a range of 0–3 points (subjective sleep quality, sleep latency, sleep duration, sleep efficiency, sleep disturbances, use of sleep medication, and daytime dysfunction), which are summated to provide the PSQI global score (range: 0–21); scores of >5 identify “poor” sleepers (Buysse et al., 1989; Curcio et al., 2013).

Sleep diaries were completed daily that included a record of bed time, sleep onset, time to fall asleep, wake-up time, get-up time, number/duration of any night awakenings, and daytime naps. Relevant metrics were calculated, including sleep onset latency, wakefulness after initial sleep onset, total sleep time, total time spent in bed, sleep efficiency (time asleep out of total time in bed, as a percentage) (Carney et al., 2012). Participants were instructed to complete the sleep diary each morning, after each night sleep. For purposes of baseline vs. light comparisons, sleep dairy information was averaged over days 1–7 (always starting on a Monday) and over days 8–21 (always starting on a Monday; *vide infra—experimental protocol*). Effects by day as well as by week (days 1–7 vs. 8–14 vs. 15–21) were evaluated.

Actigraphy was utilized to assess objective rest-activity rhythms as a proxy for sleep-wake patterns. An actigraph is a device that monitors and records body movement via an accelerometer. It has the size of a wristwatch and can be worn on the wrist, ankle or hip for several days without interfering with normal daily activities (Cellini et al., 2013). It is commonly used in conjunction with sleep diaries. In the current study we asked our participants to wear a SOMNOwatch™ actigraph (SOMNOmedics Gmbh, Randersacker, DE) on their non-dominant wrist throughout the 26 days of the study, except when showering or bathing. SOMNOwatch™ is an FDA-approved device to measure sleep parameters, which have been shown to be reliable based on a validation study against polysomnography (Dick et al., 2010). The monitors were set to record for 30-s epochs and the recordings were downloaded at the end of the recording period. Time trying to sleep and get up time were obtained from the corresponding sleep diaries and were entered into the Domino Light Panel software (v 1.4.0, SOMNOmedics Gmbh, which implements the algorithm proposed by Gorny et al., 1997). The following indices (also validated against polysomnography by Dick et al., 2010) were extracted from the software: total sleep time (minutes); sleep onset latency (time to fall asleep, minutes); sleep efficiency {100^*^[total sleep time/(bed time – get up time)]}, wake after sleep onset (time spent awake after sleep onset, minutes), number of awakenings after sleep onset. For purposes of baseline vs. light comparisons, actigraphy information was averaged over days 1–7 (always starting on a Monday) and over days 8–21 (always starting on a Monday; *vide infra—experimental protocol*). Both effects by day and by week (days 1–7 vs. 8–14 vs. 15–21) were evaluated.

### Experimental protocol and light administration

The study was conducted over 25 days; sleep-wake diaries were completed daily and an actigraph worn continuously. All participants underwent the experimental protocol over the same period of the year (from January to March) to avoid confounding from daylight saving time and/or major changes in natural environmental light conditions; each study protocol started on a Monday. The PSQI and the SF-36 questionnaires were completed on days 1 (pre-light) and 21 (end of light administration). Two separate, timed 56-h urine collections (over 2 days and 3 nights, at 4 h intervals during the day and 8 h intervals at night) were obtained on study days 3–4 (pre-light) and 22–23 (immediately after light) for the assessment of urinary 6-sulphatoxymelatonin (aMT6s; *vide infra*).

Participants were provided with a Light Box (SAD3 10000 lux—E.M.S. srl Bologna, Italy) and instructed to expose themselves to light daily for 45 min, immediately after getting-up from day 8 to day 22 (15 days in total). They were asked to place the Light Box at ~60 cm from their head, to one side, and to direct their gaze at the Light Box every 10–15 min. If they moved away from the box (for example to go to the toilet or get dressed) they were instructed to make up for the lost time at the end of the session. During the entire study period, subjects were asked to maintain regular sleep-wake schedules and avoid significant changes to their habitual consumption of coffee, tea, chocolate and alcoholic beverages. They were also asked to avoid exposure to bright light and use of mobiles/tablets in the evening and at night, as long as this did not represent a major change compared to their regular habits. Compliance was checked by phone every third day by one of the researchers.

### Urinary 6-sulfatoxymelatonin (aMT6s)

Urine samples were kept at −20°C and aMT6s concentrations measured by radioimmunoassay (Stockgrand Ltd., UK). Urine samples were analyzed in duplicate and all samples from the same participant were included in the same assay. The inter-assay coefficients of variation were 8.9% at 2.46 ± 0.22 ng/mL, 9.1% at 10.27 ± 0.93 ng/mL, and 8.9% at 20.73 ± 1.86 ng/mL. Urinary aMT6s rhythms were evaluated using cosinor analysis, which is based on the least square approximation of the time series using a cosine function with a period of 24 h (Minors and Waterhouse, 1988). The following parameters were obtained: acrophase time (time of peak aMT6s concentration, or maximum of the fitted cosinor function), mesor (mean aMT6s value for all the samples included in the cosinor analysis), amplitude (difference between the mesor and the peak aMT6s concentrations), and % rhythm (or percentage data variability accounted for by the cosine curve: 100% rhythm = all data points fall on the cosine curve, 0% rhythm = none of the data points fall on the cosine curve), which was used as a measure of robustness of the aMT6s rhythmicity. Cosinor-derived indices were calculated only if the cosinor fit was significant, based on both the likelihood of the data points fitting a straight line as opposed to a cosine curve, expressed as a p value, and on the variable % rhythm. Thus cosinor-derived indices were considered reliable if the cosinor fit was significant at the 95% level (*p* < 0.05) and the variable % rhythm equalled or exceeded 50 (Minors and Waterhouse, 1988).

### Ethics

The study was approved by the Padova University Hospital Ethics Committee (3639/AO/15, modified 09/03/2016; final approval 17/05/2016) and all participants provided written, informed consent. The study was conducted according to the Declaration of Helsinki (Hong Kong Amendment) and Good Clinical Practice (European) guidelines.

### Statistical analysis

The variables distribution was tested for normality using the Shapiro–Wilks test. Differences between normally distributed variables were examined by Student's *t*-test/one-way ANOVA; subsequent between group comparisons were performed using the Tukey HSD test. Differences between non-normally distributed variables were examined by Mann–Whitney *U*-test/Kruskal–Wallis ANOVA; subsequent between-group comparisons were performed using the median test for multiple comparisons. Pre-post light treatment differences were analyzed by repeated measures ANOVA, by group, and the Tukey test was used for *post-hoc* comparisons. The level of significance was set at *p* < 0.05 for all analyses.

## Results

### Baseline assessment

There were no significant differences in age between the three groups (healthy volunteers: 57 ± 14, PBC: 53 ± 10, cirrhosis: 57 ± 12 years) while the expected differences in sex were observed, with all enrolled PBC patients being females and patients with cirrhosis being more commonly males. There were no significant differences between the three groups in terms of quality of life (Table 1). However, absolute quality of life values were considerably lower in PBC patients (mental component) and patients with cirrhosis (physical component) compared to healthy volunteers. Diurnal preference was comparable in the three groups (Table 1).

**Table 1:** Quality of life, diurnal preference questionnaire, sleep questionnaire, and sleep diary variables (mean ± SD), by group and in relation to treatment.

		**Baseline**			**During light administration**		
		**Healthy volunteers**	**PBC**	**Cirrhosis**	**Healthy volunteers**	**PBC**	**Cirrhosis**
		**(*n* = 6)**	**(*n* = 13)**	**(*n* = 7)**	**(*n* = 6)**	**(*n* = 13)**	**(*n* = 7)**
Quality of life	SF-36 Physical Component	53.51 ± 0.71	41.42 ± 9.21	36.83 ± 12.54	53.49 ± 1.41	44.38 ± 8.24	33.01 ± 10.02
	SF-36 Mental Component	47.53 ± 14.14	35.16 ± 9.38	46.51 ± 21.78	54.52 ± 0.71	38.31 ± 10.17	47.52 ± 11.11
Diurnal preference	Horne-Östberg score	62.16 ± 11.23	55.85 ± 14.48	56.86 ± 11.07	NA	NA	NA
Subjective sleep quality (PSQI)	Component 1—Subjective sleep quality (0-3)	0.8 ± 0.4	2.1 ± 0.9^#^	1.8 ± 0.9	0.8 ± 0.4	1.1 ± 0.8^*^	1.1 ± 0.7
	Component 2–Sleep latency (0-3)	1.0 ± 0.9	2.2 ± 0.7^#^	2.0 ± 1.1	1.2 ± 1.2	1.7 ± 1.0	1.7 ± 1.2
	Component 3—Sleep duration (0-3)	0.7 ± 0.5	2.1 ± 0.8^#^	1.7 ± 0.9	0.3 ± 0.5	1.5 ± 1.1	1.3 ± 0.9
	Component 4—Sleep efficiency (0-3)	0.3 ± 0.5	1.8 ± 1.0^#^	1.3 ± 1.2	0.2 ± 0.4	1.7 ± 1.0	0.8 ± 1.1
	Component 5—Sleep disturbance (0–3)	1.2 ± 0.4	1.7 ± 0.6	1.7 ± 0.8	1.2 ± 0.4	1.5 ± 0.8	1.0 ± 0.8
	Component 6—Use of sleep medication (0–3)	0.2 ± 0.4	1.2 ± 1.2	0.7 ± 1.2	0.0 ± 0.0	0.6 ± 1.2	0.6 ± 1.0
	Component 7—Daytime dysfunction (0–3)	1.2 ± 0.7	1.9 ± 0.6	2.0 ± 0.8	0.8 ± 0.4	1.3 ± 0.5^*^	1.9 ± 0.4
	Global score (0–21)	5.3 ± 2.1	13.1 ± 4.0^#^	11.3 ± 4.8^▴^	4.5 ± 1.5	9.4 ± 3.9^*^	8.4 ± 4.5
Sleep diaries	Bed time (hh:mm)	23:58 ± 00:57	23:26 ± 00:55	23:57 ± 01:07	23:29 ± 00:49	23:03 ± 00:50	00:01 ± 00:57
	Time try to sleep (hh:mm)	00:15 ± 00:54	23:57 ± 01:01	00:11 ± 01:00	23:45 ± 00:40	23:34 ± 00:52	00:12 ± 00:53
	Sleep onset latency (min)	8.01 ± 4.81	30.82 ± 16.43	37.62 ± 57.26	6.03 ± 4.01	25.39 ± 16.63	37.7 ± 42.04
	Sleep onset (hh:mm)	00:23 ± 00:50	00:32 ± 01:12	00:48 ± 01:49	23:51 ± 00:37	23:56 ± 01:06^*^	00:51 ± 01:35
	Night awakenings (n)	1.39 ± 1.15	1.84 ± 0.85	2.83 ± 1.95	1.17 ± 0.87	1.4 ± 0.76	2.44 ± 1.81
	Wake up time (hh:mm)	06:54 ± 00:31	06:39 ± 01:22	07:26 ± 00:47	06:36 ± 00:24	06:30 ± 01:08	07:30 ± 00:52
	Get up time (hh:mm)	07:12 ± 00:37	07:21 ± 01:20	07:56 ± 00:56	06:58 ± 00:37	06:58 ± 01:07^*^	07:51 ± 00:53
	Daytime naps (n)	0.2 ± 0.4	0.4 ± 0.4	0.8 ± 0.5	0.5 ± 0.4	0.3 ± 0.4	0.7 ± 0.4
	Time spent in bed (hours)	7.2 ± 0.9	7.8 ± 1.0	8.0 ± 0.7	7.5 ± 1.1	7.9 ± 1.2	7.8 ± 0.8
	Length of sleep (hours)	6.5 ± 0.6	6.1 ± 1.1	6.7 ± 1.4	4.0 ± 1.7	5.5 ± 1.3	5.6 ± 1.3
	Sleep efficiency (%)	90.5 ± 0.8	78.3 ± 11.6	82.5 ± 14.0	91.5 ± 5.4	83.1 ± 10.2	84.6 ± 11.0

# PBC vs. healthy volunteers: p < 0.05

▴ *Cirrhosis vs. healthy volunteers: p < 0.05*.

* *PBC light treatment vs. PBC baseline: p < 0.05*.

Subjective sleep quality (PSQI Global Score) was significantly worse in patients with PBC (13.0 ± 4.0) and patients with cirrhosis (11.3 ± 4.8) compared to healthy volunteers (5.3 ± 2.1) (Table 1). Patients with PBC also fared significantly worse than healthy volunteers on single PSQI Components 1, 2, 3, and 4 (Table 1).

Sleep timing variables were comparable in the three groups (Table 1). Similarly, no significant differences were observed in actigraphy-derived indices; however, due to a technical problem with two of the actigraphs in use, complete data were available only for 2 healthy volunteers, 11 patients with PBC, and 5 patients with cirrhosis (Table 2). Finally, baseline aMT6s rhythmicity (cosinor indices) were also comparable in the three groups (Table 3); this may relate to statistical power, as on average, patients with cirrhosis showed a considerable delay (of ~3 h) in aMT6s peak compared to the other two groups (Table 3).

**Table 2:** Actigraphic sleep-wake indices (mean ± SD), by group and in relation to treatment.

**Actigraphy indices**	**Baseline**			**During light administration**		
	**Healthy volunteers**	**PBC**	**Cirrhosis**	**Healthy volunteers**	**PBC**	**Cirrhosis**
	**(*n* = 2)**	**(*n* = 11)**	**(*n* = 5)**	**(*n* = 2)**	**(*n* = 11)**	**(*n* = 5)**
Time in bed (min)	399 ± 54	437 ± 51	463 ± 38	404 ± 96	437 ± 34	465 ± 50
Total sleep time (min)	383 ± 50	403 ± 63	406 ± 38	386 ± 90	405 ± 45	428 ± 56
Sleep onset latency (min)	7 ± 0	11 ± 16	13 ± 15	7 ± 0	6 ± 8	8 ± 8
Wake after sleep onset (min)	9 ± 4	23 ± 7	44 ± 46	10 ± 5	26 ± 14	29 ± 26
Number of awakenings (n)	2.6 ± 0.3	2.4 ± 1.3	4.9 ± 2.5	2.2 ± 0.9	2.5 ± 0.7	4.1 ± 2.9
Sleep efficiency (%)	96 ± 1	92 ± 17	88 ± 11	96 ± 1	93 ± 4	92 ± 7

**Table 3:** 6-sulfatoxymelatonin (aMT6s) cosinor indices (mean ± SD), by group and in relation to treatment.

**Cosinor indices**	**Baseline**			**After light administration**		
	**Healthy volunteers**	**PBC**	**Cirrhosis**	**Healthy volunteers**	**PBC**	**Cirrhosis**
	**(*n* = 5)**	**(*n* = 7)**	**(*n* = 5)**	**(*n* = 5)**	**(*n* = 7)**	**(*n* = 5)**
Mesor (pg/ml)	12.9 ± 7.3	8.7 ± 4.5	7.0 ± 5.4	12.6 ± 5.8	8.4 ± 5.7	9.3 ± 8.9
Amplitude (pg/ml)	17.0 ± 11.2	11.4 ± 7.0	7.5 ± 8.7	14.9 ± 10.0	10.1 ± 7.9	11.0 ± 12.6
Acrophase (clock time, hh:mm)	03:33 ± 01:24	04:00 ± 01:82	07:37 ± 07:45	03:49 ± 00:45	03:48 ± 01:09	08:00 ± 07:01
Rhythm (%)	84 ± 7	72 ± 17	68 ± 13	87 ± 6	81 ± 12	81 ± 16

*These data refer only to individuals whose aMT6s cosinor fit was significant (p < 0.05 and % rhythm ≥50) both at baseline and after treatment*.

### Response to light

**Figure 1:**
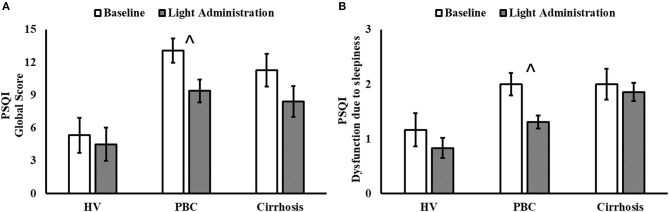
Pittsburgh Sleep Quality Index (PSQI) Global Score **(A)** and Component 7 (Dysfunction due to sleepiness; **(B)** before (white columns) and after 2 weeks of light administration (gray columns), by patient category (HV, healthy volunteers; PBC, Primary Biliary Cholangitis). **(A)** Patient category: *p* = 0.007; treatment: *p* < 0.001; patient category × treatment: *p* = 0.120; ^∧^*post-hoc* significant for PBC: *p* < 0.001. **(B)** Patient category: *p* = 0.010; treatment: *p* = 0.011; patient category × treatment: *p* = 0.280; ^∧^*post-hoc* significant for PBC: *p* = 0.021.

**Figure 2:**
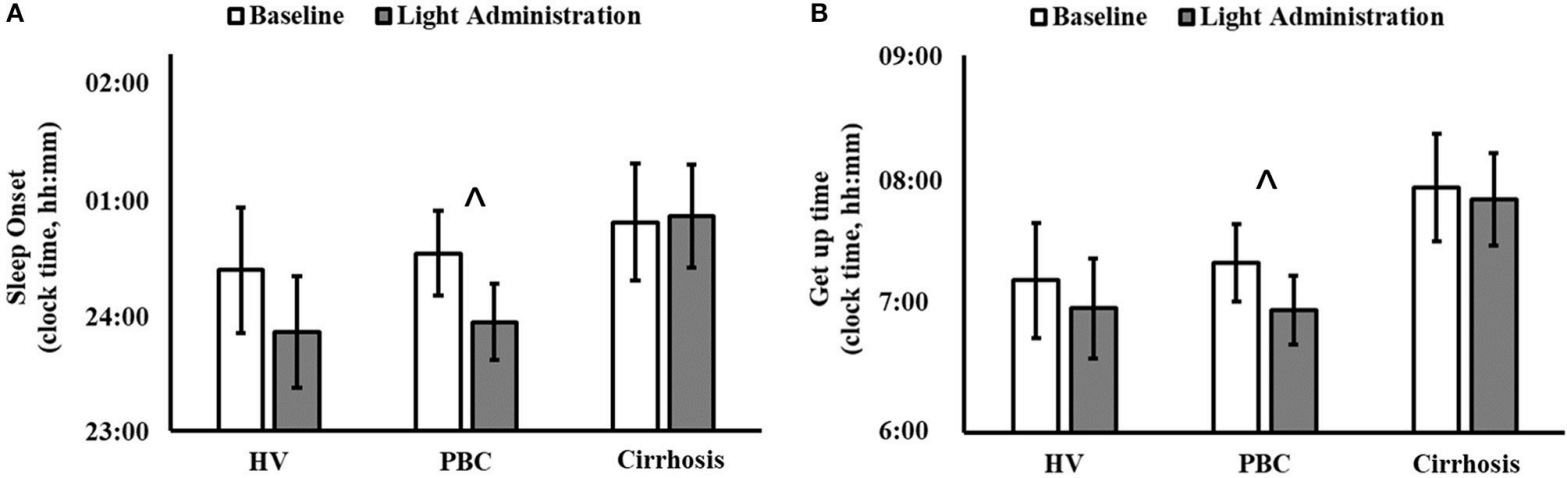
Sleep diary-based sleep onset (clock time; **A**) and get up time (clock time; **B**) before (white columns) and during light administration (gray columns), by patient category (HV, healthy volunteers; PBC, Primary Biliary Cholangitis). **(A)** Patient category: *p* = 0.500; treatment: *p* = 0.019; patient category × treatment: *p* = 0.140; ^∧^*post-hoc* significant for PBC: *p* = 0.047. **(B)** Patient category: *p* = 0.270; treatment: *p* = 0.010; patient category × treatment: *p* = 0.330; ^∧^*post-hoc* significant for PBC: *p* = 0.026.

Light exposure had a positive effect on a number of subjective sleep quality and sleep timing indices, which were significant in patients with PBC (Table 1 and Supplementary Table 1). A significant reduction in PSQI Global Score was observed in patients with PBC following light administration (9.38 ± 3.95) compared to baseline (13.08 ± 4.05, *post-hoc p* < 0.001; Table 1, Figure 1A), together with a reduction in the PSQI Component 7 (dysfunction due to sleepiness) [1.3 ± 0.5 (light) vs. 1.9 ± 0.6 (baseline), *post-hoc p* < 0.01; Table 1, Figure 1B). Furthermore, light treatment also resulted in significantly earlier sleep onset (23:56 ± 01:06 vs. 00:32 ± 01:12, *post-hoc p* = 0.047; Table 1, Figure 2A) and earlier get-up time (06:58 ± 01:07 vs. 07:21 ± 01:20, *post-hoc p* = 0.026; Table 1, Figure 2B). No significant differences were observed in actigraphy indices during light administration in any of the groups (Table 2). However, absolute values for *wake after sleep onset* decreased during light administration in patients with cirrhosis (Table 2), and the difference between baseline and treatment was significant (*p* < 0.05) when comparisons were confined to this patient group. Analyses by day and by week confirmed the averaged data analyses.

**Figure 3:**
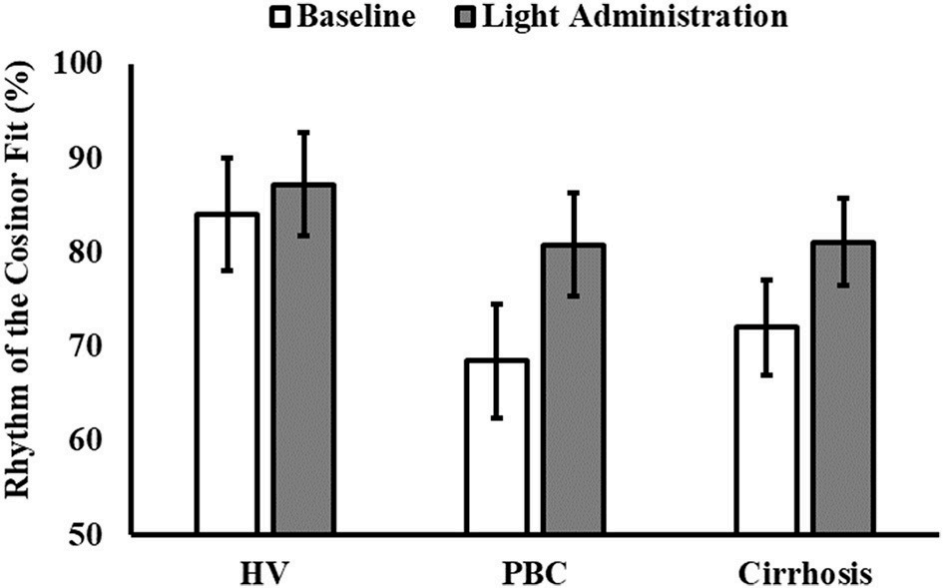
Robustness of the 6-sulphatoxymelatonin (aMT6s) rhythm (i.e., percentage data variability accounted for by the cosine curve, % rhythm) before (white columns) and after light administration (gray columns), by patient category (HV, healthy volunteers; PBC, Primary Biliary Cholangitis). Patient category: *p* = 0.250; treatment: *p* = 0.035; patient category × treatment: *p* = 0.601.

**Figure 4:**
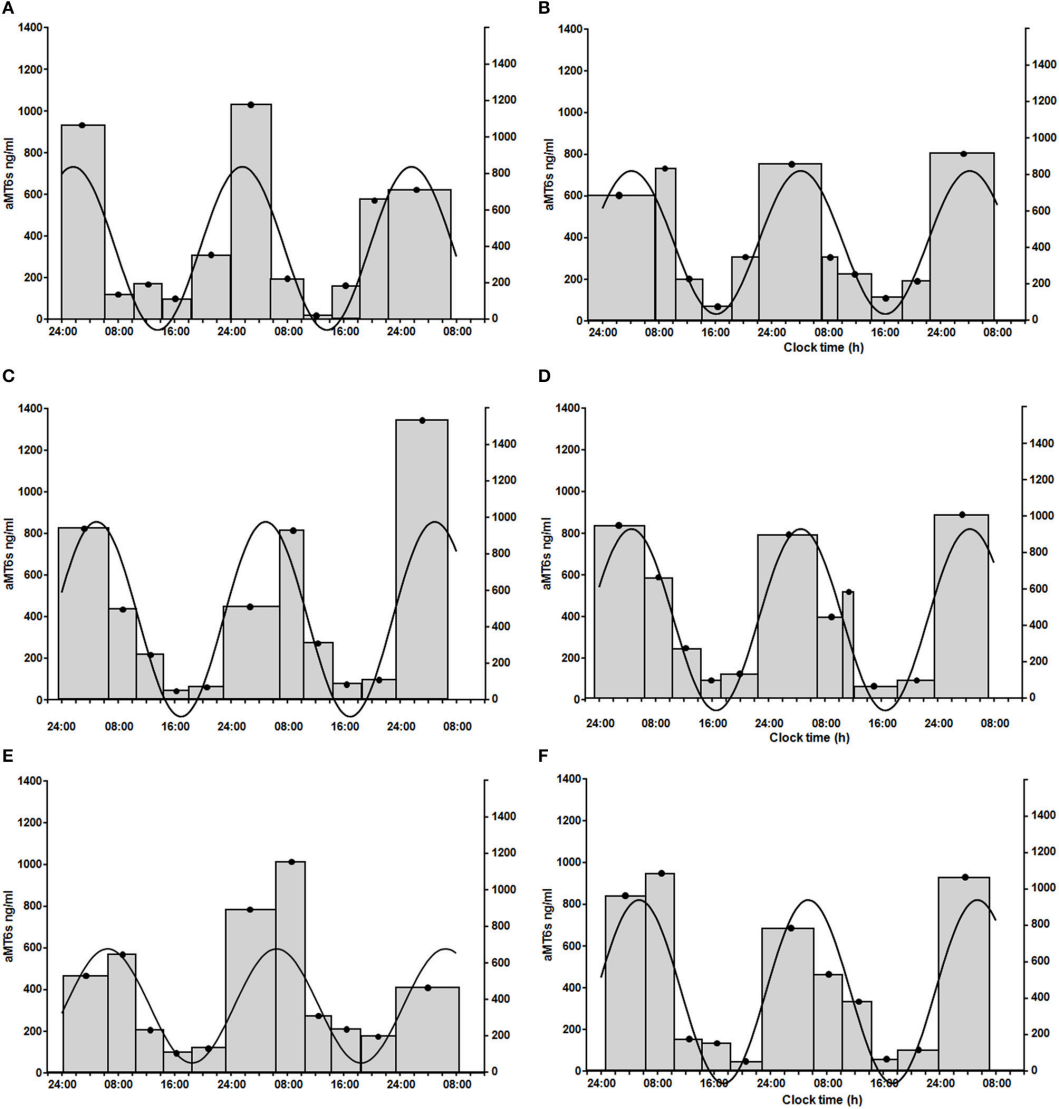
Sequential 56-h (three nights and 2 days) aMT6s excretion (ng/h, gray histograms, left y axis; width of the histogram = time between first and last bladder emptying over the collection period) in a healthy volunteer [female, 73 years of age; prior to **(A)** and after light administration **(B)**], a patient with primary biliary cholangitis [female, 65 years of age; prior to **(C)** and after light administration **(D)**], and a patient with cirrhosis [female, 50 years of age; prior to **(E)** and after light administration **(F)**]. Urinary aMT6s concentrations are high during night sleep and low during the waking day. Continuous line: fitted cosinor function, which shows the profile of urinary aMT6s concentrations over the 24-h period and allows calculation of a set of cosinor-derived aMT6s parameters (right y axis: amplitude of the cosinor fit in ng/h of aMT6s). In all three participants the cosinor fit was significant both at baseline and after light administration, the fit improving considerably after light administration. Healthy volunteer: % rhythm: 77; *p* = 0.003 at baseline **(A)** and % rhythm: 91; *p* < 0.0001 after light administration **(B)**; patient with primary biliary cholangitis: % rhythm: 59; *p* = 0.028 at baseline **(C)** and % rhythm: 95; *p* < 0.0001 after light administration **(D)**; patient with cirrhosis: % rhythm: 60; *p* = 0.026 at baseline **(E)** and % rhythm: 88; *p* < 0.001 after light administration **(F)**.

Finally, 5 healthy volunteers, 7 patients with PBC and 5 patients with cirrhosis had significant cosinor fits for their aMT6s rhythms both at baseline and after light treatment. Robustness of aMT6s rhythmicity (i.e., strength of the cosinor fit expressed as a percentage rhythm) significantly increased after light administration (Figure 3) but *post-hoc* comparisons were not significant for any of the groups (Table 3 and Supplementary Table 1). Of note, the post-light treatment improvement in cosine fits in the PBC and cirrhosis patients could not be attributed to differences in urine sample number (i.e., random variability in number of bladder emptying episodes between the pre- and post-light series) as these were comparable (*n* = 25 ± 11 at baseline and *n* = 22 ± 10 after light; *p* = 0.41). Three (one healthy volunteer, one patient with PBC, and one patient with cirrhosis) representative aMT6s histograms and pertinent cosinor fits, at baseline and after light treatment, are presented in Figure 4. In all three participants the cosinor fit was significant both at baseline and after light administration, the fit improving considerably after light administration. No significant changes were observed in the aMT6s cosinor indices mesor, amplitude and acrophase after light administration.

## Discussion

In the present study, we confirmed that non-cirrhotic patients with PBC and patients with cirrhosis of etiology other than PBC exhibit impaired sleep quality compared to healthy volunteers. In addition, we observed that a brief 15-day course of morning bright light treatment significantly improved both sleep quality and sleep timing in patients with PBC. Specifically, light exposure resulted in improved subjective night sleep quality, reduced daytime sleepiness and earlier sleep onset and get-up times. In addition, more robust aMT6s rhythmicity was observed in PBC and cirrhotic patients after light treatment. These are novel and potentially relevant observations that need to be confirmed and studied further for their potential efficacy in terms of amelioration of specific disease symptoms.

In patients with PBC, excessive daytime somnolence has been linked to fatigue but a clear association between fatigue and overall sleep quality has not yet been documented (Newton et al., 2006; Montagnese et al., 2013). In the present study, we observed amelioration of overall sleep quality, advanced sleep onset and get-up times, and a reduction in daytime sleepiness in response to bright light treatment. While fatigue was not formally assessed, it is possible to speculate that the symptoms “daytime dysfunction due to somnolence” and “fatigue” may overlap, at least to some extent, and thus hypothesize that morning bright light treatment may have some beneficial effect on fatigue, one of the most invalidating symptoms of PBC.

Using the SF-36 questionnaire, no significant differences in quality of life were observed between patients with PBC, patients with cirrhosis and healthy controls. No significant changes in quality of life were observed after light treatment either. This may be related to the limited sample size and insufficient statistical power. In addition, the SF-36 is a generic quality of life questionnaire which does not explore the quality of sleep and quality of wake domains. More specific tools, for example the PBC-40, which specifically covers the impact of fatigue and pruritus (Jacoby et al., 2005), could be utilized in future.

In the present study, patients with cirrhosis showed a very delayed aMT6s acrophase time compared to PBC and healthy volunteers, although the differences were not significant, most likely due to small sample size and considerable inter-individual variability, which has been documented before (Montagnese et al., 2015). Delayed sleep-wake (Córdoba et al., 1998), melatonin (Steindl et al., 1995, 1997; Velissaris et al., 2009; Montagnese et al., 2010), urinary aMT6s (Montagnese et al., 2009), and cortisol rhythms (Montagnese et al., 2011) have all been previously observed in these patients. Delayed melatonin, aMT6s and cortisol rhythms in this patient population are believed to be mixed in origin, i.e., due to the additive effects of impaired light sensitivity of the retinal-hypothalamic-pineal pathway (Montagnese et al., 2010), and reduced overnight melatonin clearance, especially in patients with decompensated liver disease and severe hepatic failure (Steindl et al., 1997; Montagnese et al., 2010). It is therefore interesting that the small group of patients with cirrhosis recruited in the present study, who were better compensated than those we have previously studied, also reflects the above features, with delayed aMT6s rhythms. In the present study morning bright light also had limited effects in patients with cirrhosis, which confirms a previous study of hospitalized decompensated patients with cirrhosis by our own group (De Rui et al., 2011). As this study (De Rui et al., 2011) was performed in inpatients with cirrhosis with severe hepatic failure, at the time we suspected that the limited response to light might have been due to the severity of the underlying disease, and possibly also to the added sleep-wake difficulties associated with hospitalization. By contrast, the present study appears to indicate that the response is also limited in better compensated outpatients, suggesting that alternative, cirrhosis-related mechanisms may be involved and that impaired sensitivity to light cannot be corrected simply by administering stronger/different spectrum light. The reasons why in the present study subjective sleep quality seemed to improve slightly despite the lack of an obvious aMT6s response are numerous. For example, it is possible that the observed increase in aMT6s rhythmicity, despite the lack of significant changes in aMT6s timing, may result in an improvement in sleep quality.

Our study has a number of limitations, which include: (i) its relatively small size and different sex ratio among the three study groups, largely due to the fact that PBC is a predominantly female disease whilst cirrhosis is predominantly male disease; (ii) the use of a generic quality of life questionnaire instead of specific assessment of fatigue in patients with PBC; (iii) the use of the PSQI sleep quality questionnaire, which covers sleep the month prior, thus has some degree of overlap between our baseline and post-treatment assessments; and (iv) the lack of a placebo arm, which always represents a significant problem in light treatment trials. To date, only installed placebo light facilities have been tested (Riemersma-van der Lek et al., 2008), for which a treatment effect cannot be excluded and which are not applicable in the outpatient setting. Placebo light boxes (e.g., with lower light intensity and modified spectrum) could obviously be designed but the producers' interest would be limited, and some treatment effect could not be excluded. Despite these limitations, we were able to detect a significant response to light treatment in patients with PBC, whose subjective sleep quality, sleep timing and urinary melatonin rhythmicity all improved, at least to some extent, after the course of morning bright light treatment. These results are clinically relevant and represent justification for a larger multicentre trial, with outcomes to include sleep-wake profiles, circadian hormones, fatigue, and quality of life. Future studies may also benefit from the fact that outpatient light administration tools other than light boxes have become available, e.g., light glasses (Langevin et al., 2014), which do not require the patient to stay seated in front of the light box and may improve compliance in individuals who have morning commitments.

In conclusion, should our results be confirmed in larger, possibly multicentre studies, morning bright light would represent a promising, unobtrusive and side-effect free treatment for patients with PBC with sleep-wake disturbances and daytime dysfunction.

## Author contributions

MT patient recruitment and study, data analysis, manuscript drafting; NoC patient recruitment and study, manuscript drafting; IF, CF, and FG patient recruitment and study; GF patient recruitment and study, data analysis; NiC data analysis and manuscript revision for important intellectual content; GM manuscript revision for important intellectual content; RC study funding and manuscript revision for important intellectual content; BM assays and data analysis; DS study design, data analysis, and manuscript revision for important intellectual content; AF study design, patient recruitment, and manuscript revision for important intellectual content; SM study design and funding, patient recruitment, data analysis, and manuscript drafting.

### Conflict of interest statement

The authors declare that the research was conducted in the absence of any commercial or financial relationships that could be construed as a potential conflict of interest.
