# Supplementary material for: Morning Bright Light Treatment for Sleep-Wake Disturbances in Primary Biliary Cholangitis: A Pilot Study
Source: Front Physiol. 2018 Nov 5;9:1530. doi: 10.3389/fphys.2018.01530 (PMC6230563; doi:10.3389/fphys.2018.01530)
Supplement: Supplementary file 1 [file Table_1.DOCX]

**Supplementary Table 1.** P values of the statistical analysis of differences between baseline and light treatment, by group (repeated measures ANOVA and post hoc comparisons). Please also refer to and read in parallel with Tables 1-3 and Figures 1-3.

|  | | **p value (repeated measures** **ANOVA)** | | | **p value (post hoc: Tukey test)** | | |
| --- | --- | --- | --- | --- | --- | --- | --- |
|  |  | **Patient category** | **Treatment** | **Patient category*Treatment** | **Healthy volunteers** | **PBC** | **Cirrhosis** |
| **Subjective Sleep Quality**  **(PSQI)** | PSQI Global Score (0-21) | **0.007** | *<* **0.001** | 0.120 | 0.960 | *<* **0.001** | 0.057 |
|  | Component 7 Daytime dysfunction (0-3) | **0.010** | **0.011** | 0.280 | 0.789 | **0.021** | 0.990 |
| **Sleep Diaries** | Sleep onset (hh:mm) | 0.500 | **0.019** | 0.140 | 0.426 | **0.047** | 0.999 |
|  | Wake up time (hh:mm) | 0.180 | 0.250 | 0.420 |  |  |  |
|  | Get up time (hh:mm) | 0.270 | **0.010** | 0.330 | 0.739 | **0.026** | 0.987 |
|  | Naps (n) | 0.100 | 0.920 | 0.480 |  |  |  |
|  | Awakenings (n) | 0.100 | **0.017** | 0.800 | 0.958 | 0.189 | 0.626 |
|  | Sleep onset latency (min) | 0.150 | 0.120 | 0.700 |  |  |  |
|  | Sleep efficiency (%) | 0.140 | 0.060 | 0.430 |  |  |  |
| **Actigraphic Indices** | Time in bed (min) | 0.164 | **0.040** | 0.345 | 0.410 | 0.999 | 0.564 |
|  | Total sleep time (min) | 0.738 | 0.896 | 0.990 |  |  |  |
|  | Sleep onset latency (min) | 0.945 | 0.254 | 0.802 |  |  |  |
|  | Wake after sleep onset (min) | 0.343 | 0.480 | 0.163 |  |  |  |
|  | Number of awakenings (n) | 0.077 | 0.269 | 0.387 |  |  |  |
|  | Sleep efficiency (%) | 0.590 | 0.228 | 0.266 |  |  |  |
| **Cosinor Indices** | Mesor (pg/ml) | 0.427 | 0.416 | 0.204 |  |  |  |
|  | Amplitude (pg/ml) | 0.219 | 0.593 | 0.659 |  |  |  |
|  | Achrophase (clock time, hh:mm) | 0.219 | 0.593 | 0.659 |  |  |  |
|  | Rhythm (%) | 0.250 | **0.035** | 0.601 | 0.995 | 0.427 | 0.563 |

*Bold typeface marks significant p values. Post hoc test p values were only obtained if ANOVA was significant (p < 0.05).*
